# Progressive Neurodegeneration or Endogenous Compensation in an Animal Model of Parkinson's Disease Produced by Decreasing Doses of Alpha-Synuclein

**DOI:** 10.1371/journal.pone.0017698

**Published:** 2011-03-07

**Authors:** James B. Koprich, Tom H. Johnston, Philippe Huot, M. Gabriela Reyes, Maria Espinosa, Jonathan M. Brotchie

**Affiliations:** Toronto Western Research Institute, Toronto Western Hospital, University Health Network, Toronto, Ontario, Canada; Johns Hopkins, United States of America

## Abstract

The pathological hallmarks of Parkinson's disease (PD) are degeneration of dopamine (DA) neurons of the substantia nigra (SN) and the presence of alpha-synuclein (α-syn)-rich Lewy bodies in DA cells that remain. To model these aspects of the disease, we previously showed that high titer (5.1×10exp12 gp/ml) AAV1/2 driven expression of A53T α-syn in the SN of rats caused nigrostriatal pathology including a loss of DA neurons, but also with toxicity in the GFP control group. In the current study, we evaluate the effects of two lower titers by dilution of the vector (1∶3 [1.7×10exp12] and 1∶10 [5.1×10exp11]) to define a concentration that produced pathology specific for α-syn. In GFP and empty vector groups there were no behavioural or post-mortem changes at 3 or 6 weeks post-administration at either vector dose. Dilution of the AAV1/2 A53T α-syn (1∶3) produced significant paw use asymmetry, reductions in striatal tyrosine hydroxylase (TH), and increases in DA turnover at 3 weeks in the absence of overt pathology. By 6 weeks greater evidence of pathology was observed and included, reductions in SN DA neurons, striatal DA, TH and DA-transporter, along with a sustained behavioural deficit. In contrast, the 1∶10 AAV1/2 A53T α-syn treated animals showed normalization between 3 and 6 weeks in paw use asymmetry, reductions in striatal TH, and increased DA turnover. Progression of dopaminergic deficits using the 1∶3 titer of AAV1/2 A53Tα-syn provides a platform for evaluating treatments directed at preventing and/or reversing synucleinopathy. Use of the 1∶10 titer of AAV1/2 A53T α-syn provides an opportunity to study mechanisms of endogenous compensation. Furthermore, these data highlight the need to characterize the titer of vector being utilized, when using AAV to express pathogenic proteins and model disease process, to avoid producing non-specific effects.

## Introduction

Parkinson's disease (PD) is a neurodegenerative disorder characterized clinically by rigidity, slowness of movement and tremor and neuropathologically by a severe loss of dopamine (DA) neurons of the substantia nigra (SN) and with Lewy bodies in the majority of those that remain. Lewy bodies are proteinaceous intra-neuronal inclusions that are largely composed of alpha-synuclein (α-syn) and their presence is often necessary for the final diagnosis of PD [1], [2], [3], [4]. The normal function of α-syn has yet to be fully characterized; however, mounting evidence supports its role as a synaptic protein involved with vesicular release of neurotransmitters (including DA) [5], [6], [7], [8], [9], [10], [11], [12]. Over-expression studies have also implicated a normal physiological role for α-syn in axonal transport [13] and in mechanisms of autophagy [14], [15], [16], [17].

Triplication, duplication or mutations (A53T, A30P, E46K) in the gene encoding α-syn, SNCA1, produce familial forms of PD [18], [19], [20], [21], [22]. Not long after these discoveries, transgenic mice [16], [23], [24], [25], [26], [27] and viral vector rodent and primate models over-expressing α-syn were reported [13], [28], [29], [30], [31], [32], [33]. While these transgenic mice have proven useful in advancing understanding of how high levels of α-syn interfere with normal cellular function, they fail to model the nigrostriatal pathology and basal ganglia-based motor features of PD. Viral vector models were able to overcome this by targeting the basal ganglia directly through injection into the SN and have been shown repeatedly to produce a progressive nigrostriatal degeneration along with motor impairments. However, to date viral vector-based models show variability in degrees of pathology and onset to expression of behavioural impairments [13], [30], [31], [32], [34].

In an effort to develop a new viral vector-based model of PD that could consistently deliver nigrostriatal pathology and relevant behavioural deficits in a relatively short period of time (more amendable to *in vivo* drug evaluation) we have previously used a high titer formulation of chimeric AAV1/2 human A53T α-syn (5.1×10^12^ gp/ml) and examined tissues 3 weeks after injection into the SN [35]. We found that overexpression of A53T α-syn produced insoluble aggregates along with dopaminergic degeneration. However, in control studies where the same vector, at the same titer, was used to express GFP we found evidence of SN neuronal loss. Although this AAV1/2 GFP-induced cell loss was significantly less than with the AAV1/2 A53T α-syn cases, it clearly showed that we could not attribute all the effects to A53T α-syn *per se*. Since the empty vector controls showed no evidence of neurodegeneration we concluded that expression of a protein from this high concentration of vector was sufficiently burdensome to be toxic to SN DA neurons.

In the current study, we have formulated the same AAV1/2 vectors to assess whether lower titer preparations might produce degeneration specific to A53T α-syn. We report that a 1∶3 dilution (1.7×10^12^ gp/ml) produces a progressive degeneration along with a behavioural impairment that occurs over the course of 6 weeks, while a 1∶10 dilution (5.1×10^11^ gp/ml) produces signs of dopaminergic dysfunction and a behavioural impairment at 3 weeks that are shown to reverse by 6 weeks. In both conditions, no sign of GFP toxicity is observed.

## Results

### Co-localization and forebrain expression of proteins delivered by AAV1/2 vectors

**Figure 1:**
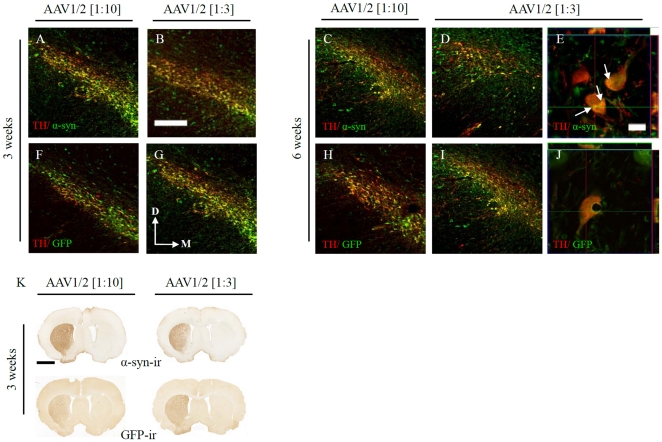
Transgene expression in the substantia nigra and striatum. AAV1/2 delivered to the substantia nigra (SN) as two concentrations (1∶3, 1.7×10^12^ gp/ml and 1∶10, 5.1×10^11^ gp/ml) resulted in expression of human α-synuclein (A–E) or GFP (F–J) in the vast majority SN TH-ir neurons and transport of transgenes to the striatum (K). Confocal imaging and orthogonal views of collected Z-stacks confirmed that α-synuclein accumulated (white arrows in G) within SN TH-ir neurons (G). Scale bar in panel A is 200 µm and applies to panels A–D, F–I. Scale bar in panel G is 20 µm and applies to panels G and J. Scale bar in panel K is 1 mm.

Delivery of human A53T α-syn or GFP using AAV1/2 vectors resulted in expression in >95% of dopamine (DA) neurons within the anatomical boundaries of the substantia nigra (SN). Figure 1 shows that both concentrations of viral vector produced similar degrees of co-localization with DA neurons (using tyrosine hydroxylase as a marker; TH) of the SN by 3 weeks, which was maintained through 6 weeks, indicating that the subsequent results are not due to the number of DA neurons being infected by AAV1/2 particles, but rather the levels of expression within the transduced neurons.

Accumulations of human α-syn were observed in transfected SN DA neurons and co-expressed with TH (Figure 1E). Expression of α-syn or GFP in the striatum was prevalent, by 3 weeks, throughout its anatomical boundaries regardless of the dose of AAV1/2 used (Figure 1K). Transgene expression in the SN and striatum remained at similar levels 6 weeks post vector injection (data not shown).

### Forelimb use in the cylinder test

**Figure 2:**
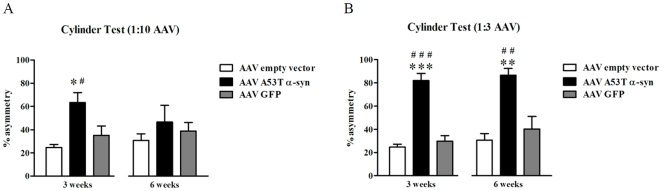
Forelimb asymmetry in the cylinder test. Three weeks following injection of AAV1/2 A53T α-synuclein a significant asymmetry in forelimb use is present in rats exposed to either titer of vector (1∶10, 5.1×10^11^ gp/ml [panel A] or 1∶3, 1.7×10^12^ gp/ml [panel B]). At six weeks however the rats injected with the 1∶10 dilution recovered on this test (A), while the rats injected with the 1∶3 dilution continued to show a behavioural impairment (B). *^,^**^,^*** *c.f.* empty vector, *P*<0.05, 0.01, and 0.001 respectively; ^#,##,###^ *c.f.* GFP, *P*<0.05, 0.01, and 0.001 respectively.

Animals that received either the 1∶10 (F_2,20_ = 5.55, *P* = 0.012) or 1∶3 (F_2,21_ = 36.05, *P*<0.0001) concentration of AAV1/2 A53T α-syn showed a deficit in the use of their paw contralateral to the side of vector injection 3 weeks post surgery compared to GFP (1∶10, *P*<0.05; 1∶3, *P*<0.001) and empty vector (EV) (1∶10, *P*<0.05; 1∶3, *P*<0.001) controls. This was represented by a 39% and a 57% increase forelimb use asymmetry in 1∶10 and 1∶3 AAV1/2 α-syn injected rats, respectively, compared to EV controls. Six weeks following vector injection, animals injected with 1∶10 AAV1/2 A53T α-syn showed a recovery in function compared to controls (*P*>0.05), while the animals in the 1∶3 administered group demonstrated a persistent asymmetry in forelimb paw use (F_2,20_ = 9.6, *P* = 0.001; *post-hoc*, *cf.* GFP or EV, both *P*<0.01). See Figure 2.

### Cell number in the substantia nigra

**Figure 3:**
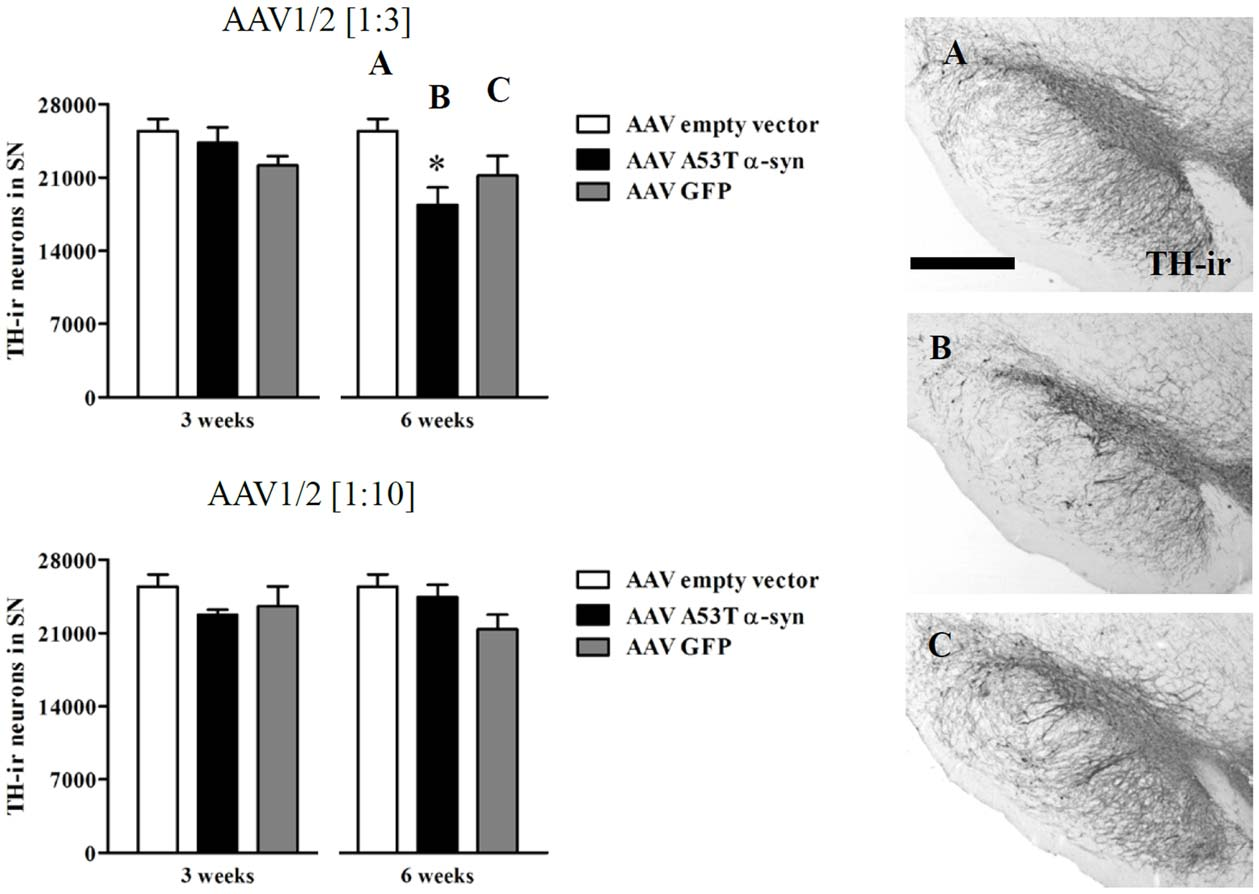
Dopamine neuron loss in the substantia nigra. Stereological counting of TH-ir neurons in the substantia nigra (SN) showed that AAV1/2 A53T α-synuclein (1∶3, 1.7×10^12^ gp/ml) reduces the number of dopamine neurons 6 weeks following intra-nigral injection, while quantification at 3 weeks or when using the 1∶10 dilution (5.1×10^11^ gp/ml) at either timepoint, showed no evidence of neuron loss. Panels A, B and C represent significant comparisons found in the AAV1/2 [1∶3] graphs. Scale bar in panel A is 1000 µm. * *P*<0.05 *c.f.* empty vector.

Three weeks following delivery of vectors coding for GFP, or α-syn and an empty vector, no difference in the number of TH-immunoreactive (ir) cells in the SN was evident (*P*>0.05). In contrast, at the 6 week timepoint, there was a significant difference in the number of TH-ir cells in the SN when compared across all three groups in the 1∶3 condition (Figure 3A–C) (F_2,14_ = 4.37, *P*<0.05). *Post-hoc* analysis revealed that animals that received the 1∶3 AAV1/2 A53T α-syn showed a significant decrease (by 28%) in TH-ir cells compared to EV controls (*P*<0.05) and no significant difference to GFP controls (*P*>0.05). There were no significant differences among conditions in the 1∶10 dilution at the 6 week timepoint (*P*>0.05). See Figure 3.

### Expression of tyrosine hydroxylase and axonal morphology of nigrostriatal fibers in the striatum

**Figure 4:**
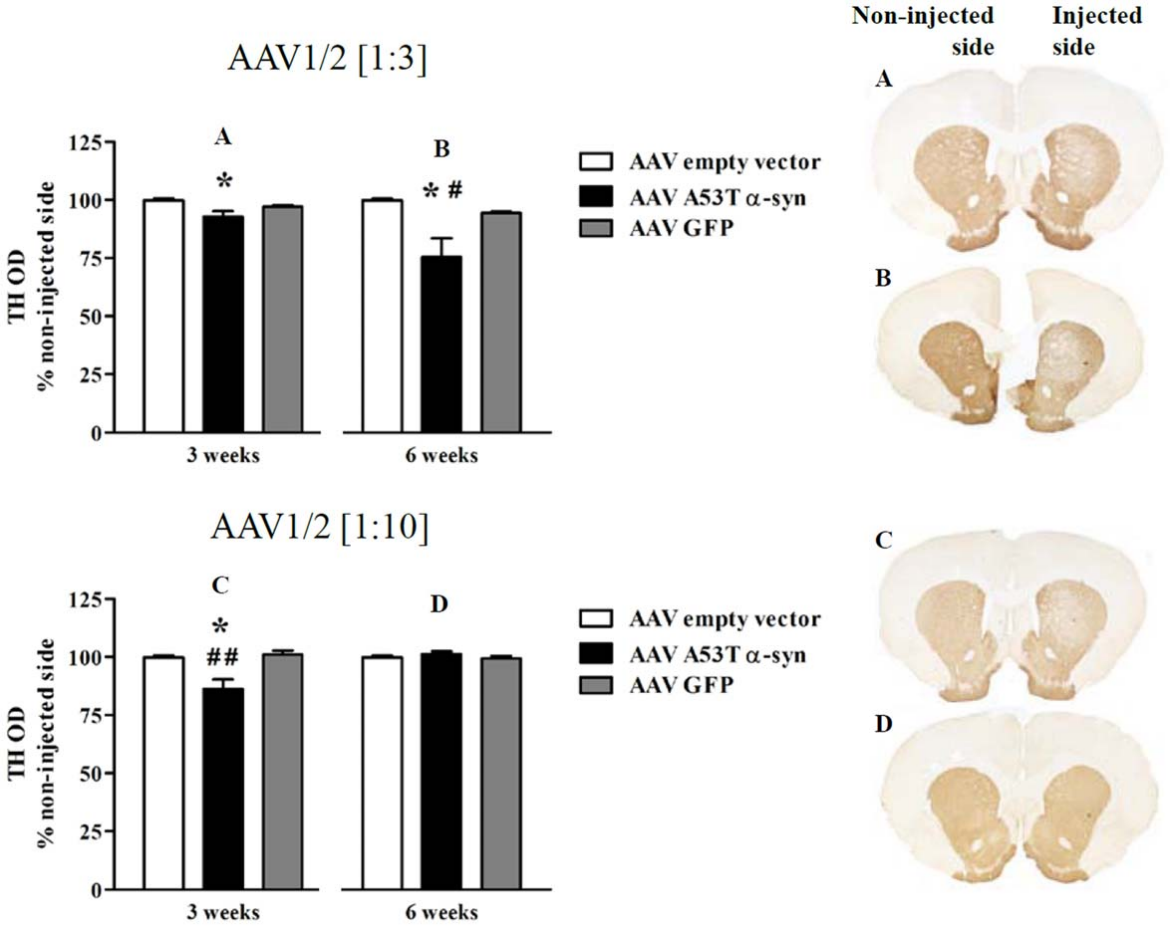
Reduced striatal tyrosine hydroxylase expression in AAV1/2 A53T α-synuclein injected rats. Three weeks following intra-nigral injection of both titers of AAV1/2 A53T α-syn (1∶3, 1.7×10^12^ gp/ml and 1∶10, 5.1×10^11^ gp/ml) there was a significant reduction in the expression of striatal tyrosine hydroxylase (TH). By six weeks the reduction in striatal TH had recovered in the 1∶10 condition (D), while in the 1∶3 condition, a greater loss in TH was observed (B). Panels A, B, C and D represent significant comparisons found in the graphs. * *P*<0.05 *c.f.* empty vector, ^#^ *c.f.* GFP.

Expression of TH in the striatum, as measured by optical density, was shown to be significantly reduced at the 3 week timepoint in both the 1∶3 (by 7% from EV) (F_2,14_ = 4.77, *P*<0.05; *post-hoc*, *c.f.* EV, *P*<0.05) and 1∶10 A53T α-syn conditions (by 13% from EV) (F_2,14_ = 8.93, *P*<0.01; *post-hoc*, *c.f.* EV, *P*<0.05, *c.f.* GFP, *P*<0.01) (Figure 4A,C). In contrast, by 6 weeks, the expression of TH normalized in the 1∶10 condition (*P*>0.05) compared to EV and GFP controls and was shown to be further reduced in the 1∶3 condition (24.5% from EV) (F_2,14_ = 6.57, *P*<0.01; *post-hoc*, *c.f.* GFP or EV, both *P*<0.05) (Figure 4B,D).

**Figure 5:**
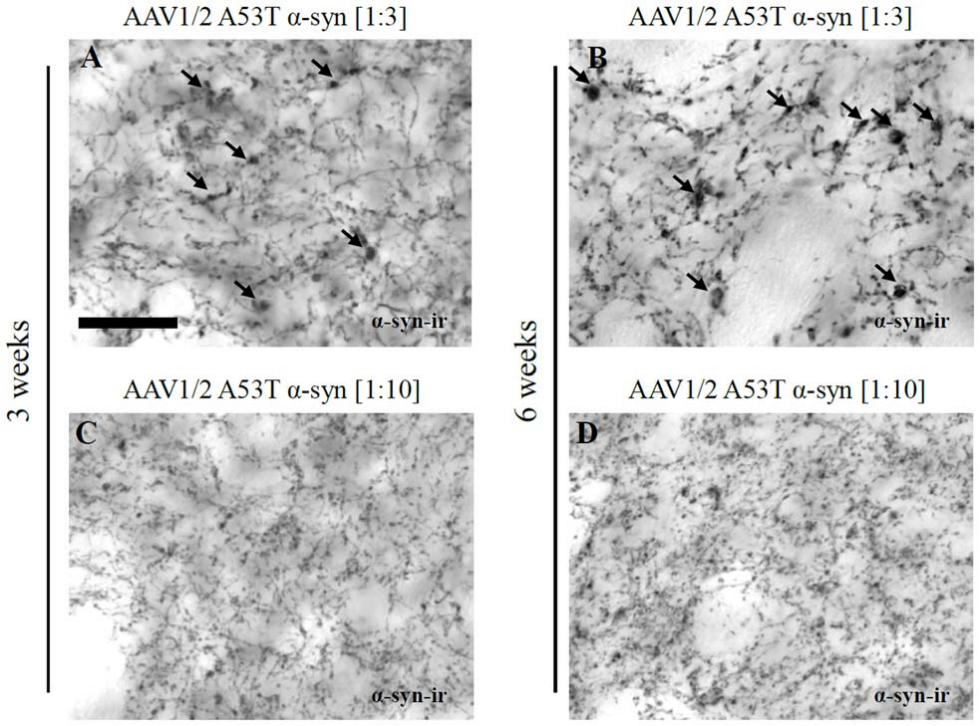
Dystrophic axon morphology in rats injected with AAV1/2 A53T α-synuclein. Delivery of AAV1/2 A53T alpha-synuclein (1∶3, 1.7×10^12^ gp/ml) to the substantia nigra produces dystrophic and bulging neurites in the striatum 3 and 6 weeks later with increasing severity (A,B). Injection of the 1∶10 dilution (1∶10, 5.1×10^11^ gp/ml) of AAV1/2 AAV1/2 A53T α-syn also showed signs of abnormal axonal architecture, although not to the same degree (C,D). Arrows point to evidence of human alpha-synuclein positive dystrophic neurites; scale bar is 50 µm.

The reduction in striatal TH expression was accompanied by the appearance of abnormal axon morphology. Using antibodies directed against human α-syn, axonal architecture in the striatum was examined. The presence of dystrophic neurites were evident in the 1∶3 AAV1/2 A53T α-syn injected animals by 3 weeks and were shown to be more severe or pronounced after 6 weeks (Figure 5A,B), while the degree of abnormal axonal morphology in the 1∶10 AAV1/2 A53T α-syn injected animals was minimal or absent at both the 3 and 6 week timepoints (Figure 5C,D).

### Dopamine transporter and neurochemistry in the striatum

**Figure 6:**
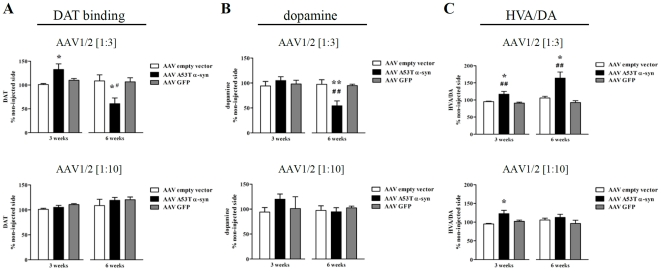
Striatal changes in dopamine transporter and neurochemistry following intra-nigral injection of AAV1/2 A53T α-synuclein. Three weeks post AAV1/2 A53T α-syn injection of the 1∶3 dilution there was a significant rise in dopamine transporter (DAT) followed 3 weeks later by a significant reduction (A). No changes in striatal DAT was observed using the 1∶10 dilution of AAV1/2 A53T α-syn at either timepoint (A). Dopamine loss was seen only in the 1∶3 dilution of AAV1/2 A53T α-syn and only after 6 weeks of exposure (B). Dopamine turnover (HVA/DA) was significantly elevated in both the 1∶3 and 1∶10 AAV1/2 A53T α-syn conditions, 3weeks post vector injection. By 6 weeks however, DA turnover had returned to normal in the 1∶10 AAV1/2 A53T α-syn group and had increased further in the 1∶3 AAV1/2 A53T α-syn group (C). *^,^** *c.f.* empty vector, *P*<0.05 and 0.01, respectively; ^#,##^ *c.f.* GFP, *P*<0.05 and 0.01, respectively.

Delivery of human A53T α-syn (1∶3 dilution) to the SN produced a 31% increase in striatal DAT binding, measured by autoradiography, 3 weeks post injection (F_2,15_ = 4.94, *P* = 0.02; *post-hoc*, *c.f.* EV, *P*<0.05). However, at the 6 week timepoint DAT binding was shown to be significantly reduced by 48% compared to levels in the EV condition (F_2,18_ = 5.61, *P* = 0.013; *post-hoc*, *c.f.* EV or GFP, both *P*<0.05) (Figure 6A). No significant alterations in DAT binding were seen in the 1∶10 A53T α-syn or in either concentration of GFP.

Using HPLC to quantify total DA levels and its metabolites in the striatum we found that expression of human A53T α-syn produced significant alterations. Firstly, DA was unchanged in both the 1∶3 and 1∶10 dilutions at the 3 week timepoint, however at 6 weeks DA was significantly reduced by 43% in the 1∶3 condition (F_2,21_ = 9.55, *P*<0.01; *post-hoc*, *c.f.* EV or GFP, both *P*<0.01) and remained unchanged in the 1∶10 condition (Figure 6B). The turnover of DA as measured by the ratio, HVA/DA, was significantly increased by 26% in the 1∶10 dilution (F_2,20_ = 5.04, *P*<0.05; *post-hoc*, *c.f.* EV, *P*<0.05) and by 22% in the 1∶3 dilution at 3 weeks compared to EV controls (F_2,21_ = 8.49, *P* = 0.002; *post-hoc*, *c.f.* EV, *P*<0.05; *c.f.* GFP, *P*<0.01). At the 6 week timepoint, DA turnover was further increased in the 1∶3 condition compared to EV controls (now a 58% increase) (F_2,19_ = 10.53, *P*<0.001; *post-hoc*, *c.f.* EV, *P*<0.001 or *c.f.* GFP *P*<0.05), while in contrast DA turnover was restored to control levels (GFP and EV) in the 1∶10 condition (Figure 6C). Neither the GFP nor EV groups had any impact on striatal DA neurochemistry as compared to their respective contralateral side or when compared to each other.

## Discussion

In the vast majority of Parkinson's disease cases, irrespective of etiology, is the presence of intraneuronal Lewy bodies composed primarily of alpha-synuclein (α-syn) within the substantia nigra (SN). In the current study, we showed that delivery of human A53T α-syn to the rat SN using AAV1/2 results in a behavioural deficit along with key elements of nigro-striatal degeneration associated with PD and in a time- and dose-dependent manner.

Previously we have shown that delivery of this same vector, albeit at a higher titer, 5.1×10^12^ gp/ml, produced a loss of SN TH-ir and NeuN-ir cells within 3 weeks [35]. In that study, α-syn deposits in the surviving SN dopamine (DA) neurons were insoluble, as evidenced by resistance to proteinase K digestion. Accompanying this were reductions in TH-ir and DAT in the striatum and a marked presence of dystrophic neurites compared to empty vector controls. However, in that previous study, we showed that high titer AAV1/2 GFP also produced, though to a lesser extent, degeneration in the SN (loss of TH-ir and NeuN-ir cells) without signs of pathology at the level of the striatum [35]. In that study, human A53T α-syn could not be solely accountable for the pathology produced, suggesting that there likely exists a toxic effect of overloading SN TH-ir neurons with a protein. In light of these data, we embarked on the current study to examine the effects of 2 lower titers of the AAV1/2 to define a concentration of viral particles that produced nigro-striatal pathology that could be attributed to human A53T α-syn, and not to an effect of protein overload.

We report that a dilution of 1∶3 (final titer of 1.7×10^12^ gp/ml) achieved this goal although following a longer time course than the undiluted vector stock. Thus, 3 weeks following SN injection of AAV1/2 we found that α-syn accumulated in SN TH-ir neurons in the absence of cell loss and a moderate degree of dystrophic axons were apparent in the striatum. Furthermore, striatal TH was significantly reduced, dopamine turnover (HVA/DA) and dopamine transporter (DAT) were increased and an asymmetry in forelimb paw use was present in the cylinder test. However, six weeks following vector injection this model shows mounting evidence of nigrostriatal pathology, including further reductions in striatal TH, a more severe presence of dystrophic neurites, a reduction in striatal DA and DAT, a greater increase in DA turnover, and a sustained behavioural deficit compared to both empty vector and AAV1/2 GFP control groups. On the measure of SN TH-ir cell counts however, AAV1/2 A53T α-syn was only significantly different from empty vector controls and not to AAV1/2 GFP (AAV1/2 GFP injected rats were not significantly different from those that received the empty vector). Overall, these data are consistent with those reported previously [13], [30], [32], [33], [35], [36]. Thus, overexpression of α-syn by AAV injected into the SN consistently has produced signs of dopaminergic dysfunction along the nigrostriatal path. The time it takes (3–16 weeks) and the severity of dopaminergic deficits produced varies (cell loss ranging from 30–60%) between studies and likely depends on the combination of serotypes, promoters and viral titers utilized. In the current study we show that 28% of TH-ir SN neurons are lost compared to empty vector controls measured 6 weeks post AAV1/2 injection. What has been less consistent is the expression of behavioural deficits in these models and the apparent reliance on significant cell death as an underlying factor. In the current study, a clear behavioural deficit (cylinder test) is observed by 3 weeks post AAV1/2 A53T α-syn injection. Of interest, the first appearance of a behavioural deficit, at 3 weeks, was without the requisite of cell death. Such a finding has not previously been reported; indeed previous studies report cell loss without a behavioural deficit [13], [32], [34]. This finding has been replicated in our hands across experiments within and between vector preparations (data not shown).

Of considerable interest was the finding of a behavioural impairment at 3 weeks in the absence of a reduction in striatal DA levels or SN cell numbers, while in the presence of moderate dystrophic axonal morphology and a modest reduction in striatal TH, in animals that received the 1∶3 dilution of the AAV1/2 A53T α-syn. In traditional toxin based rodent models of PD (6-OHDA and MPTP) motor deficits are not encountered until at least 50% of the SN DA neurons are lost and with that a significant reduction in striatal DA (∼80%). This finding further suggests that α-syn plays a significant role at the synaptic terminal, involved with DA and its handling at the level of the vesicles antecedent to overt pathology. It has recently been reported that α-syn participates in managing the *release pool* of vesicles in DA terminals and that levels of synaptic proteins VAMP-2 and synapsin-1 are significantly altered after exposure to increasing levels of α-syn [12]. Although not directly tested in our current study, it is possible that in our model, A53T α-syn acts to interfere with vesicular release of DA and this underlies the behavioural phenotype. In this scenario, DA levels would appear normal (as seen with whole tissue HPLC), although DA release necessary for normal motor behaviour might be compromised. In order to begin to address this hypothesis, future studies will need to be conducted that, for example, quantified DA release (e.g. *in vivo* microdialysis) and/or measured a panel of synaptic proteins involved in vesicle regulation. Evidence that α-syn inhibits neurotransmitter release has been recently reported [10], [37]. In Chung et al (2009) it was reported, in rats, that AAV2 overexpression of A53T α-syn produced a slow progressive pathology that was related to alterations in axonal transport, synaptic proteins, and induction of neuroinflammation [13]. These data further support the contention that overexpression of α-syn produces a phase of axonal or terminal stress prior to overt degeneration that can have significant functional consequences.

Of additional interest is the finding that DAT binding, in rats injected with 1∶3 AAV1/2 A53T α-syn, was significantly elevated (31%) 3 weeks post injection and prior to signs of degeneration. At this timepoint there was also a reduction in striatal TH, an increase in DA turnover, and a deficit in contralateral paw use. Elevated DAT levels may represent an attempted compensatory response aimed at maintaining normal levels of DAT in light of reduced DA terminals. This response however fails to normalize terminal function and potentially contributes to the motor deficit observed (i.e. excessive DAT may contribute to reduced synaptic DA and result in motor dysfunction). In support of this hypothesis, transgenic mice that overexpress DAT (by 30%) have been shown to release 40% less DA and uptake it at a rate 50% greater compared to controls [38]. Taken together, if α-syn interferes with DA release [10], [12] and initial DA fibre loss results in above normal DAT levels (seen here), the net result could be less synaptic DA available for transmission and thereby abnormal motor behaviour. As the disease model progresses to the six week timepoint, however, fibers are increasingly lost (measured by TH and DAT) and the prototypical nigrostriatal pathology becomes apparent (DA neuron loss in SN and reduced striatal DA). Therefore, at the 3 week timepoint, it is likely that the behavioural deficits are a result of A53T α-syn-induced dysfunction of a relatively anatomically intact nigrostriatal pathway, while by 6 weeks, deficits are a combination of an ongoing A53T α-syn induced impairment of DA function and frank degeneration of the nigrostriatal system. It is not known whether the behavioural deficits produced here are reversible by administration of L-DOPA (L-3,4-dihydroxyphenylalanine) or a DA agonist. Future studies will investigate this.

The 1∶3 dilution used here we consider to be a model of progressive degeneration. We showed that, over time, A53T α-syn resulted in (1) decreasing levels of striatal TH, (2) increasing rates of DA turnover, (3) progressively more severe dystrophic axonal architecture, (4) a rise and subsequent fall in striatal DAT, (5) a loss of TH-ir cells in the SN and (6) a sustained behavioural impairment. This implementation of the model is ideally suited for studying potential neuroprotective, disease modifying or neurorestorative treatments. For example, delivery of a treatment close in time to the delivery of the viral vector, could evaluate potential to prevent formation of α-syn aggregates, measured at 3 weeks, and subsequently verify that this resulted in neuroprotection, measured at 6 weeks when degeneration is expected in controls. In a disease modifying paradigm, one could commence delivery of the treatment at 3 weeks, a timepoint when there is aggregated α-syn in the absence of cell loss, and evaluate its efficacy 3 weeks later and directly correlate outcome with levels of α-syn. This paradigm in particular will allow for an evaluation of treatments to target various species of α-syn. Thirdly, a neurorestorative design can be envisaged, whereby the treatment is administered after degeneration had already begun, i.e. at the 6 week timepoint and evaluate outcome several weeks later.

The study also defined a model that produced signs of neuronal stress that over time self-corrected. Thus, delivery of the 1∶10 titer, 5.1×10^11^ gp/ml AAV1/2 A53T α-syn, resulted in a behavioural deficit, an increase in DA turnover, and a reduction in striatal levels of TH when measured at 3 weeks. By 6 weeks, each of these measures had returned to normal. Delivery of this dose of A53T α-syn via this particular AAV1/2 is thus a model to study endogenous compensation and could prove promising in discovering targets to modify disease course.

In conclusion, we have developed a viral vector rat model of Parkinson's disease, based on overexpression of human A53T α-syn that is suitably titered to produce a specific and progressive degeneration of the nigrostriatal system. Two models emerged from this work, one that is progressively degenerative and another that demonstrates endogenous compensation. The former will be used to investigate new therapeutics directly targeting alpha-synuclein and its associated pathology and the latter to understand the mechanisms by which compensation can occur which has potential to generate new therapeutic targets.

## Materials and Methods

### Animals and vector delivery

Using standard stereotaxic procedures, 160 female Sprague Dawley rats (∼280 g, Charles River) were injected intranigrally with either empty AAV1/2 vector, AAV1/2 expressing GFP, or AAV1/2 expressing human A53T alpha-synuclein, under isoflurane/oxygen anesthesia. In each case, a single 2 µl injection of viral vector was delivered to the SN at a rate of 0.2 µl/min according to the following coordinates from bregma: AP, −5.2 mm; ML, −2.0 mm; DV, −7.5 mm (from skull at bregma) using a microinjector (Stoelting, Kiel, WI) and according to the atlas of Paxinos and Watson [39]. The AAV1/2 stock concentration for each vector was 5.1×10^12^ genomic particles (gp)/ml. Two concentrations of this stock, for both AAV1/2 GFP and AAV1/2 A53T α-syn were used, 1.7×10^12^ gp/ml (1∶3 dilution) and 5.1×10^11^ gp/ml (1∶10 dilution). Animals were housed in pairs in a temperature controlled environment (20°C), kept on regular 12 hr light/dark cycle (lights on 0630) and allowed food and water *ad libitum*. All procedures were conducted under an approved IACUC (University Health Network, protocol 1738.6) in accordance with guidelines and regulations set by the Canadian Council on Animal Care.

### Vectors

Adeno associated vectors (AAV) of a 1/2 serotype were designed such that expression was driven by a chicken beta actin (CBA) promoter hybridized with the cytomegalovirus (CMV) immediate early enhancer sequence. In addition, a woodchuck post-transcriptional regulatory element (WPRE) and a bovine growth hormone polyadenylation sequence (bGH-polyA) were incorporated to further enhance transcription following transduction. AAV1/2 is a chimeric vector where the capsid expresses AAV1 and AAV2 serotype proteins in a 1∶1 ratio and uses the inverted terminal repeats (ITRs) from AAV2 according to the following scheme: CMV/CBA promoter––human A53T alpha-synuclein or GFP––WPRE-bGH-polyA––ITR [40]. The vectors were produced by GeneDetect Ltd., Auckland, New Zealand. Viral titers were determined by quantitative PCR (Applied Biosystems 7900 QPCR) with primers directed to the WPRE region, thus representing the number of functional physical particles of AAV in the solution containing the genome to be delivered. Full details of vector design can be found in Koprich et al., 2010 [35].

### Cylinder Test

Three and six weeks following AAV1/2 injection, spontaneous forepaw use was evaluated using the cylinder test. Briefly, rats with their right paws marked black were placed into a glass cylinder in front of 2 mirrors and they were video recorded. Videos were scored *post-hoc* by an observer blinded to the treatment conditions. For each rearing by the animal it was recorded whether it used its left or right forepaw to touch the inner surface of the glass cylinder. Scoring was conducted over a period of 6 min. A minimum of 20 total touches was considered acceptable for inclusion in the analysis. The final data are presented as percent asymmetry (disparity between left [contralateral to the injection] and right paw use [ipsilateral to the lesion]), which was derived from the following equation according to Wishaw and Kolb (2005): [(% right paw use)−(% left paw use)/(% right paw use)+(% left paw use)] * 100 [41].

### Post-mortem measures

#### Immunohistochemistry

Three and six weeks after AAV1/2 injection, animals were administered an overdose of pentobarbital (1.0 ml of 240 mg/ml, i.p.) and killed by exsanguination by transcardial perfusion with saline followed by 4% paraformaldehyde. Brains were then removed and processed for immuno-labeling. Brains were sectioned frozen in the coronal plane at a thickness of 40 µm on a sliding microtome (Leica Microsystems Inc., Richmond Hill, ON) and 6 series of sections were stored in cryoprotectant (30% glycerol, 30% ethoxyethanol, 40% PBS). A single series of sections was processed for visualization of tyrosine hydroxylase (TH) via the biotin-labelled antibody procedure. Briefly, following several washes in a PBS solution containing 0.2% Triton X-100 (PBS-T), endogenous peroxidase was quenched in a 3% hydrogen peroxide solution and background staining was then inhibited in a 10% normal goat serum/2% bovine serum albumin solution. Tissue was then incubated with primary antibodies overnight: rabbit anti-TH antibody (1∶1000, Pel-Freez, Rogers, AR), mouse anti-NeuN antibody (1∶200, Millipore, Billerica, MA). After three washes in PBS-T, sections were sequentially incubated in biotinylated goat anti-rabbit or mouse IgG (1∶300; Vector, Burlingame, CA) for 1 h and the Elite avidin-biotin complex (ABC Kits; Vector, Burlingame, CA) for 1 h separated by three washes in PBS. Immunostaining was visualized following a reaction with 3,3-diaminobenzidine (Vector, Burlingame, CA). Sections were then mounted on glass slides, allowed to dry, dipped into dH_2_0, dehydrated through graded alcohols (70%, 95%, 100%), cleared in xylenes, and coverslipped with DPX mounting medium (Electron Microscopy Sciences, Hatfield, PA).

In the striatum, the degree of TH expression was assessed using optical density (OD) measurements of immuno-labelled tissue (ImageJ, version 1.43u). Images were captured under uniform light conditions using a slide scanner (Myer Instruments, Houston,TX). Three anatomical levels were assessed (pre-commissural, commissural, and post-commissural) and for each level the contralateral striatum was also assessed. Final values represent the average of the 3 anatomical levels taken as a percentage of the average of each corresponding contralateral level.

Immunofluorescence (Confocal microscopy, Zeiss Axioplan with LSM510META) to reveal TH (1∶1000, Pel-Freez, Rogers, AR) and human alpha-synuclein (1∶500, Zymed, San Francisco, CA) or GFP (1∶2000, Abcam, Cambridge, MA) simultaneously was conducted. Images were taken throughout the Z-axis to confirm co-localization of α-syn and GFP within individual TH neurons.

#### Stereology

TH stained sections of the SN were used for stereological estimation of dopamine neuron numbers using optical fractionator from the Stereo Investigator software package (v. 7, MBF Biosciences, Williston, VT). The user was blinded to group assignment by coded slides. Nine sections spanning the entire anterior/posterior extent of the SN, each separated by 240 µm (1/6 series), were used for counting. All TH-ir neurons of the SN (pars compacta and pars reticulata) were included within each contour, of each section. Parameters used for TH stereological counting were grid size, 300 µm×300 µm; counting frame, 80 µm×80 µm, and 2 µm guard zones. Tissue thickness was determined by the user at each counting site. All final values represent *estimated total by number weighted section thickness* and were only included if their Gunderson coefficient of error (m = 1) was less than 0.09.

#### Autoradiography

The levels of striatal DAT were assessed by [^125^I]-RTI-121 binding autoradiography in sections prepared from fresh-frozen tissue. Briefly, thawed slides were placed in binding buffer (2×15 min, room temperature) containing 50 mM Tris, 120 mM NaCl and 5 mM KCl. Sections were then placed in the same buffer containing 50 pM [^125^I]-RTI-121 (Perkin-Elmer, specific activity 2200 Ci/µmol) for 120 min at 25°C to determine total binding. Non-specific binding was defined as that observed in the presence of 100 µM GBR 12909 (Tocris Bioscience). All slides were then washed (4×15 min) in ice-cold binding buffer, rinsed in ice-cold distilled water and air-dried. Together with [^125^I]-microscale standards (Amersham) slides were then apposed to autoradiographic film (Kodak) and left for 2 days at 4°C before developing. Autoradiograms were analysed using MCID software (Image Research Inc, Ontario, Canada). Densitometric analysis of 3 striata from each animal was carried out whereby a reference curve of c.p.m. versus optical density was calculated from β-emitting [^14^C] micro-scale standards and used to quantify the intensity of signal as nCi/g. Background intensity was subtracted from each reading. Data were then expressed as mean ± s.e.m. signal intensity for each treatment group. Non-specific binding was calculated in the same way and subtracted from the total to give to give specific binding. Non-specific binding was found to account for <1% of total binding.

#### Catecholamine quantification

Tissue pieces were sent to CMN/KC Neurochemistry Core Lab in Vanderbilt University for HPLC analysis. The brain sections were homogenized in 200–750 ul of 0.1 M TCA, which contains 10^−2^ M sodium acetate, 10-4 M EDTA and 10.5% methanol (pH 3.8) using a tissue dismembrator (Fisher Scientific). Samples were spun in a microcentrifuge at 10000 g for 20 minutes. The supernatant was removed and stored at −80 degrees. The pellet was saved for protein analysis. Supernatant was then thawed and spun for 20 minutes. Catecholamines were determined by a specific HPLC assay utilizing an Antec Decade II (oxidation: 0.5) electrochemical detector operated at 33°C. Samples of the supernatant were injected using a Water 717+ autosampler onto a Phenomenex Nucleosil (5u, 100A) C18 HPLC column (150×4.60 mm). Analytes were eluted with a mobile phase consisting of 89.5% 0.1 M TCA, 10^−2^ M sodium acetate, 10^−4^ M EDTA and 10.5% methanol (pH 3.8). Solvent was delivered at 0.8 ml/min using a Waters 515 HPLC pump. Using this HPLC solvent, analytes were observed in the following order: DOPAC, dopamine, and HVA. HPLC control and data acquisition were managed by Waters Empower software. Total protein for each sample was determined using the Peirce BCA protein assay. Values of catecholamines are expressed as ng analyte/mg total protein.

### Statistical Analysis

For all statistical comparisons we first used a 1-way ANOVA, with significance set at *P*<0.05. If ANOVA was significant, all *post-hoc* tests were conducted using Tukey's Multiple Comparison test. Software used to conduct statistical analyses and graph all data was Prism v. 5.02 (GraphPad, La Jolla, CA, USA).
